# INTERMUSCULAR ADIPOSITY: A NOVEL RISK FACTOR FOR COGNITIVE DECLINE IN A BIRACIAL COHORT OF OLDER ADULTS

**DOI:** 10.1093/geroni/igac059.1310

**Published:** 2022-12-20

**Authors:** Caterina Rosano, Anne Newman, Xiaonan Zhu, Adam Santanasto, Bret Goodpaster, Iva Miljkovic

**Affiliations:** University of Pittsburgh, Pittsburgh, Pennsylvania, United States; University of Pittsburgh, Pittsburgh, Pennsylvania, United States; University of Pittsburgh, Pittsburgh, Pennsylvania, United States; School of Public Health, University of Pittsburgh, Oakmont, Pennsylvania, United States; AdventHealth, Orlando, Florida, United States; University of Pittsburgh, Pittsburgh, Pennsylvania, United States

## Abstract

Skeletal muscle and brain health both decline with age. Poorer skeletal muscle characteristics may be early markers of cognitive decline. The Health ABC study obtained repeated measures of thigh intermuscular adiposity via CT (IMAT, Years 1 and 6) and Mini-Mental State Exam (MMSE, Years 1 through 10), in 1634 adults (69-79 years, 48% women, 35% black). Linear mixed effects models accounted for change (Years 1 and 6) in weight, muscle (strength, area), and adiposity characteristics (abdominal subcutaneous, visceral, total fat mass) and for dementia risk factors (education, APOE4, diabetes, hypertension, physical activity). IMAT increased by 0.97 cm2/year (SD:1.16), and MMSE declined by 0.4 points/year (0.02). Each SD of IMAT corresponded to a MMSE decline of 0.22 points/year (p<0.0001), similar in adjusted models and stratified by race or gender. Aging-related IMAT increase may be a novel predictor of cognitive decline beyond traditional risk factors, with potential implications for muscle-brain cross talk.

